# *N*′-[(*E*)-2-Chloro-5-nitro­benzyl­idene]-2-nitro­benzohydrazide

**DOI:** 10.1107/S1600536810026723

**Published:** 2010-07-14

**Authors:** Huanyu Liu

**Affiliations:** a School of Chemistry and Chemical Engineering, Guangdong Pharmaceutical University, Zhongshan 528453, People’s Republic of China

## Abstract

In the title compound, C_14_H_9_ClN_4_O_5_, the mol­ecule exists in a *trans* geometry with respect to the methyl­idene unit. The dihedral angle between the two substituted benzene rings is 62.7 (2)°. In the crystal, inversion dimers linked by pairs of N—H⋯O hydrogen bonds generate *R*_2_^2^(8) loops.

## Related literature

For a related structure and background references, see: Liu (2010 ▶).
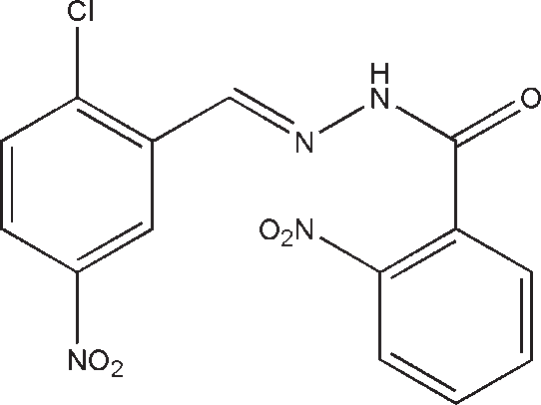


## Experimental

### 

#### Crystal data

C_14_H_9_ClN_4_O_5_
*M* *_r_* = 348.70
Triclinic, 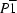

*a* = 7.432 (3) Å
*b* = 9.296 (4) Å
*c* = 12.404 (5) Å
α = 77.621 (5)°
β = 87.674 (6)°
γ = 76.271 (5)°
*V* = 813.1 (6) Å^3^
*Z* = 2
Mo *K*α radiation
μ = 0.27 mm^−1^
*T* = 298 K
0.20 × 0.18 × 0.17 mm

#### Data collection

Bruker SMART CCD diffractometer
Absorption correction: multi-scan (*SADABS*; Sheldrick, 1996 ▶) *T*_min_ = 0.949, *T*_max_ = 0.956
4610 measured reflections
2863 independent reflections
1878 reflections with *I* > 2σ(*I*)
*R*_int_ = 0.018

#### Refinement

*R*[*F* ^2^ > 2σ(*F* ^2^)] = 0.069
*wR*(*F* ^2^) = 0.207
*S* = 1.00
2863 reflections
220 parameters
1 restraint
H atoms treated by a mixture of independent and constrained refinement
Δρ_max_ = 1.26 e Å^−3^
Δρ_min_ = −0.30 e Å^−3^

### 

Data collection: *SMART* (Bruker, 1998 ▶); cell refinement: *SAINT* (Bruker, 1998 ▶); data reduction: *SAINT*; program(s) used to solve structure: *SHELXS97* (Sheldrick, 2008 ▶); program(s) used to refine structure: *SHELXL97* (Sheldrick, 2008 ▶); molecular graphics: *SHELXTL* (Sheldrick, 2008 ▶); software used to prepare material for publication: *SHELXTL*.

## Supplementary Material

Crystal structure: contains datablocks global, I. DOI: 10.1107/S1600536810026723/hb5545sup1.cif

Structure factors: contains datablocks I. DOI: 10.1107/S1600536810026723/hb5545Isup2.hkl

Additional supplementary materials:  crystallographic information; 3D view; checkCIF report

## Figures and Tables

**Table 1:** Hydrogen-bond geometry (Å, °)

*D*—H⋯*A*	*D*—H	H⋯*A*	*D*⋯*A*	*D*—H⋯*A*
N2—H2⋯O3^i^	0.90 (1)	2.05 (1)	2.937 (3)	170 (4)

Symmetry code: (i) 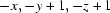.

**Fig. 1.**
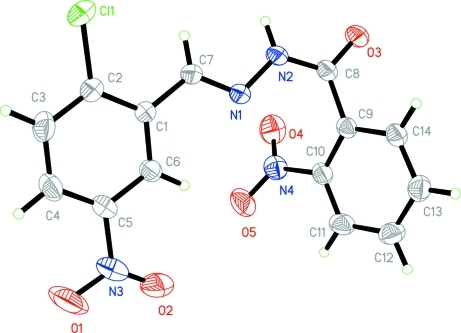
Molecular structure of (I) with 30% probability displacement ellipsoids.

**Fig. 2.**
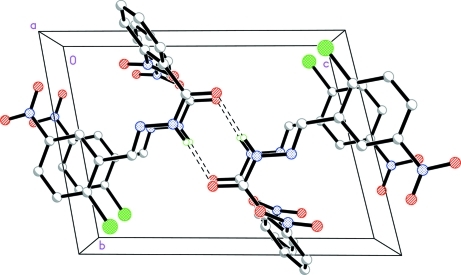
Packing structure of (I), viewed along the a axis. Hydrogen bonds are shown as dashed lines.

## supplementary crystallographic information

### Comment

Recently, the author has reported a hydrazone compound (Liu, 2010). As a further study on these compounds, in the present work, a new hydrazone compound is reported.

In the title compound (Fig. 1), the hydrazone molecule exists in a *trans* geometry with respect to the methylidene unit. The dihedral angle between the two substituted benzene rings is 62.7 (2)°. The O1/N3/O2 nitro plane forms a dihedral angle of 3.7 (2)° with the C1-C6 benzene ring. The O4/N4/O5 nitro plane forms a dihedral angle of 33.9 (2)° with the C9-C14 benzene ring. In the crystal structure, adjacent two molecules are linked through two N—H···O hydrogen bonds (Table 1) to form a dimer (Fig. 2).

### Experimental

2-Chloro-5-nitrobenzaldehyde (1.0 mmol, 185 mg) and 2-nitrobenzohydrazide (1.0 mmol, 181 mg) were mixed in 50 mL methanol. The mixture was stirred at ambient temperature for 2 h and filtered. Colorless blocks of (I) were formed by slow evaporation of the filtrate for 5 d.

### Refinement

The amino hydrogen atom was located in an electronic density map and refined isotropically, with the N—H distance restrained to 0.90 (1)Å. Other hydrogen atoms were placed in calculated positions, with C—H = 0.93 Å, and refined as riding with U_iso_(H) = 1.2U_eq_(C). The structure contains solvent accessible VOIDS of 70 Å^3^, which might accord a disordered water molecule.

### Crystal data

**Table:**

C_14_H_9_ClN_4_O_5_	*Z* = 2
*M**_r_* = 348.70	*F*(000) = 356
Triclinic, *P*1	*D*_x_ = 1.424 Mg m^−^^3^
Hall symbol: -P 1	Mo *K*α radiation, λ = 0.71073 Å
*a* = 7.432 (3) Å	Cell parameters from 1380 reflections
*b* = 9.296 (4) Å	θ = 2.3–25.3°
*c* = 12.404 (5) Å	µ = 0.27 mm^−^^1^
α = 77.621 (5)°	*T* = 298 K
β = 87.674 (6)°	Block, colorless
γ = 76.271 (5)°	0.20 × 0.18 × 0.17 mm
*V* = 813.1 (6) Å^3^	

### Data collection

**Table:**

Bruker SMART CCD diffractometer	2863 independent reflections
Radiation source: fine-focus sealed tube	1878 reflections with *I* > 2σ(*I*)
graphite	*R*_int_ = 0.018
ω scans	θ_max_ = 25.5°, θ_min_ = 3.7°
Absorption correction: multi-scan (*SADABS*; Sheldrick, 1996)	*h* = −8→8
*T*_min_ = 0.949, *T*_max_ = 0.956	*k* = −11→11
4610 measured reflections	*l* = −14→15

### Refinement

**Table:**

Refinement on *F*^2^	Primary atom site location: structure-invariant direct methods
Least-squares matrix: full	Secondary atom site location: difference Fourier map
*R*[*F*^2^ > 2σ(*F*^2^)] = 0.069	Hydrogen site location: inferred from neighbouring sites
*wR*(*F*^2^) = 0.207	H atoms treated by a mixture of independent and constrained refinement
*S* = 1.00	*w* = 1/[σ^2^(*F*_o_^2^) + (0.1395*P*)^2^] where *P* = (*F*_o_^2^ + 2*F*_c_^2^)/3
2863 reflections	(Δ/σ)_max_ < 0.001
220 parameters	Δρ_max_ = 1.26 e Å^−^^3^
1 restraint	Δρ_min_ = −0.30 e Å^−^^3^

### Special details

**Table:**

Geometry. All esds (except the esd in the dihedral angle between two l.s. planes) are estimated using the full covariance matrix. The cell esds are taken into account individually in the estimation of esds in distances, angles and torsion angles; correlations between esds in cell parameters are only used when they are defined by crystal symmetry. An approximate (isotropic) treatment of cell esds is used for estimating esds involving l.s. planes.
Refinement. Refinement of F^2^ against ALL reflections. The weighted R-factor wR and goodness of fit S are based on F^2^, conventional R-factors R are based on F, with F set to zero for negative F^2^. The threshold expression of F^2^ > 2sigma(F^2^) is used only for calculating R-factors(gt) etc. and is not relevant to the choice of reflections for refinement. R-factors based on F^2^ are statistically about twice as large as those based on F, and R- factors based on ALL data will be even larger.

### Fractional atomic coordinates and isotropic or equivalent isotropic displacement parameters (Å^2^)

**Table:**

	*x*	*y*	*z*	*U*_iso_*/*U*_eq_	
Cl1	0.26862 (17)	0.10161 (9)	0.90359 (9)	0.0893 (4)	
N1	0.0650 (3)	0.5612 (3)	0.73068 (18)	0.0489 (6)	
N2	0.0178 (4)	0.5714 (3)	0.62253 (19)	0.0572 (7)	
N3	0.2718 (5)	0.6264 (5)	1.1036 (3)	0.0751 (9)	
N4	0.1665 (4)	0.8525 (3)	0.6682 (3)	0.0640 (8)	
O1	0.3378 (7)	0.5946 (5)	1.1976 (3)	0.1348 (14)	
O2	0.2136 (6)	0.7508 (4)	1.0530 (3)	0.1165 (13)	
O3	−0.1109 (4)	0.7087 (3)	0.46232 (17)	0.0702 (7)	
O4	0.2419 (4)	0.8013 (3)	0.5910 (3)	0.0858 (8)	
O5	0.2494 (4)	0.8607 (3)	0.7502 (3)	0.0919 (9)	
C1	0.2110 (4)	0.4041 (3)	0.8957 (2)	0.0463 (7)	
C2	0.2716 (5)	0.2577 (3)	0.9592 (3)	0.0577 (8)	
C3	0.3337 (6)	0.2313 (4)	1.0669 (3)	0.0731 (10)	
H3	0.3750	0.1327	1.1068	0.088*	
C4	0.3341 (5)	0.3520 (4)	1.1146 (3)	0.0669 (9)	
H4	0.3737	0.3363	1.1872	0.080*	
C5	0.2751 (4)	0.4952 (4)	1.0530 (2)	0.0534 (7)	
C6	0.2128 (4)	0.5243 (3)	0.9448 (2)	0.0503 (7)	
H6	0.1726	0.6235	0.9056	0.060*	
C7	0.1508 (4)	0.4296 (3)	0.7812 (2)	0.0516 (7)	
H7	0.1752	0.3494	0.7448	0.062*	
C8	−0.0717 (4)	0.7033 (3)	0.5596 (2)	0.0513 (7)	
C9	−0.1414 (4)	0.8379 (3)	0.6106 (2)	0.0466 (7)	
C10	−0.0355 (4)	0.9083 (3)	0.6632 (2)	0.0515 (7)	
C11	−0.1089 (5)	1.0291 (4)	0.7086 (3)	0.0655 (9)	
H11	−0.0330	1.0713	0.7442	0.079*	
C12	−0.3001 (6)	1.0897 (4)	0.7013 (3)	0.0679 (9)	
H12	−0.3530	1.1742	0.7304	0.081*	
C13	−0.4087 (5)	1.0227 (4)	0.6508 (3)	0.0640 (9)	
H13	−0.5362	1.0615	0.6465	0.077*	
C14	−0.3317 (4)	0.8988 (3)	0.6062 (2)	0.0539 (7)	
H14	−0.4082	0.8550	0.5726	0.065*	
H2	0.059 (5)	0.490 (3)	0.593 (3)	0.080*	

### Atomic displacement parameters (Å^2^)

**Table:**

	*U*^11^	*U*^22^	*U*^33^	*U*^12^	*U*^13^	*U*^23^
Cl1	0.1262 (9)	0.0423 (5)	0.0944 (8)	−0.0049 (5)	−0.0208 (6)	−0.0162 (4)
N1	0.0592 (14)	0.0462 (13)	0.0415 (12)	−0.0080 (11)	−0.0069 (10)	−0.0131 (10)
N2	0.0807 (18)	0.0518 (14)	0.0384 (13)	−0.0060 (13)	−0.0138 (12)	−0.0157 (11)
N3	0.085 (2)	0.097 (3)	0.0616 (18)	−0.0388 (19)	0.0027 (15)	−0.0368 (18)
N4	0.0637 (18)	0.0618 (17)	0.0698 (18)	−0.0260 (14)	−0.0047 (15)	−0.0076 (14)
O1	0.209 (4)	0.147 (3)	0.076 (2)	−0.064 (3)	−0.030 (2)	−0.052 (2)
O2	0.184 (4)	0.0693 (19)	0.104 (2)	−0.020 (2)	−0.030 (2)	−0.0391 (18)
O3	0.1005 (17)	0.0660 (14)	0.0406 (12)	−0.0051 (12)	−0.0208 (11)	−0.0157 (10)
O4	0.0632 (16)	0.102 (2)	0.0902 (19)	−0.0215 (14)	0.0062 (14)	−0.0149 (16)
O5	0.0805 (18)	0.100 (2)	0.103 (2)	−0.0297 (15)	−0.0357 (15)	−0.0219 (16)
C1	0.0505 (16)	0.0424 (15)	0.0448 (15)	−0.0063 (12)	−0.0044 (12)	−0.0107 (12)
C2	0.0676 (19)	0.0459 (16)	0.0570 (18)	−0.0073 (14)	−0.0055 (14)	−0.0107 (14)
C3	0.089 (2)	0.060 (2)	0.0555 (19)	−0.0032 (18)	−0.0098 (17)	0.0065 (16)
C4	0.077 (2)	0.078 (2)	0.0410 (16)	−0.0118 (18)	−0.0085 (15)	−0.0075 (16)
C5	0.0533 (17)	0.069 (2)	0.0445 (16)	−0.0210 (15)	0.0005 (12)	−0.0176 (14)
C6	0.0578 (17)	0.0491 (16)	0.0453 (15)	−0.0152 (13)	−0.0042 (13)	−0.0092 (12)
C7	0.0641 (18)	0.0454 (16)	0.0440 (15)	−0.0049 (13)	−0.0057 (13)	−0.0145 (12)
C8	0.0601 (17)	0.0535 (17)	0.0407 (15)	−0.0123 (14)	−0.0090 (13)	−0.0099 (13)
C9	0.0554 (17)	0.0442 (15)	0.0399 (14)	−0.0148 (13)	−0.0071 (12)	−0.0032 (11)
C10	0.0583 (18)	0.0464 (16)	0.0503 (16)	−0.0168 (13)	−0.0103 (13)	−0.0043 (12)
C11	0.087 (3)	0.0514 (18)	0.066 (2)	−0.0263 (18)	−0.0113 (17)	−0.0159 (15)
C12	0.091 (3)	0.0481 (18)	0.064 (2)	−0.0095 (17)	−0.0033 (18)	−0.0172 (15)
C13	0.063 (2)	0.064 (2)	0.0571 (18)	−0.0017 (16)	−0.0046 (15)	−0.0086 (16)
C14	0.0613 (19)	0.0528 (17)	0.0459 (15)	−0.0110 (14)	−0.0114 (13)	−0.0073 (13)

### Geometric parameters (Å, °)

**Table:**

Cl1—C2	1.740 (3)	C3—H3	0.9300
N1—C7	1.277 (4)	C4—C5	1.364 (5)
N1—N2	1.378 (3)	C4—H4	0.9300
N2—C8	1.341 (4)	C5—C6	1.388 (4)
N2—H2	0.898 (10)	C6—H6	0.9300
N3—O2	1.180 (5)	C7—H7	0.9300
N3—O1	1.232 (5)	C8—C9	1.496 (4)
N3—C5	1.481 (4)	C9—C14	1.392 (4)
N4—O4	1.217 (4)	C9—C10	1.394 (4)
N4—O5	1.235 (4)	C10—C11	1.353 (4)
N4—C10	1.467 (4)	C11—C12	1.398 (5)
O3—C8	1.241 (3)	C11—H11	0.9300
C1—C6	1.384 (4)	C12—C13	1.371 (5)
C1—C2	1.398 (4)	C12—H12	0.9300
C1—C7	1.460 (4)	C13—C14	1.376 (5)
C2—C3	1.383 (5)	C13—H13	0.9300
C3—C4	1.377 (5)	C14—H14	0.9300
			
C7—N1—N2	115.0 (2)	C1—C6—H6	120.3
C8—N2—N1	121.2 (2)	C5—C6—H6	120.3
C8—N2—H2	120 (3)	N1—C7—C1	120.4 (2)
N1—N2—H2	118 (3)	N1—C7—H7	119.8
O2—N3—O1	124.6 (4)	C1—C7—H7	119.8
O2—N3—C5	120.1 (3)	O3—C8—N2	119.6 (3)
O1—N3—C5	115.3 (4)	O3—C8—C9	120.4 (3)
O4—N4—O5	124.2 (3)	N2—C8—C9	119.7 (2)
O4—N4—C10	117.8 (3)	C14—C9—C10	116.3 (3)
O5—N4—C10	118.0 (3)	C14—C9—C8	117.0 (3)
C6—C1—C2	117.7 (3)	C10—C9—C8	126.7 (3)
C6—C1—C7	121.1 (3)	C11—C10—C9	123.4 (3)
C2—C1—C7	121.2 (2)	C11—C10—N4	117.4 (3)
C3—C2—C1	122.0 (3)	C9—C10—N4	119.2 (3)
C3—C2—Cl1	117.9 (2)	C10—C11—C12	119.1 (3)
C1—C2—Cl1	120.0 (2)	C10—C11—H11	120.5
C4—C3—C2	119.6 (3)	C12—C11—H11	120.5
C4—C3—H3	120.2	C13—C12—C11	119.0 (3)
C2—C3—H3	120.2	C13—C12—H12	120.5
C5—C4—C3	118.6 (3)	C11—C12—H12	120.5
C5—C4—H4	120.7	C12—C13—C14	121.0 (3)
C3—C4—H4	120.7	C12—C13—H13	119.5
C4—C5—C6	122.8 (3)	C14—C13—H13	119.5
C4—C5—N3	119.3 (3)	C13—C14—C9	121.2 (3)
C6—C5—N3	117.9 (3)	C13—C14—H14	119.4
C1—C6—C5	119.3 (3)	C9—C14—H14	119.4

### Hydrogen-bond geometry (Å, °)

**Table:**

*D*—H···*A*	*D*—H	H···*A*	*D*···*A*	*D*—H···*A*
N2—H2···O3^i^	0.90 (1)	2.05 (1)	2.937 (3)	170 (4)

Symmetry codes: (i) −*x*, −*y*+1, −*z*+1.
